# External validation of the five domains of negative symptoms: Focus on cognition, functional capacity, and real-world functioning

**DOI:** 10.1192/j.eurpsy.2023.2478

**Published:** 2023-12-15

**Authors:** Giulia M. Giordano, Francesco Sanmarchi, Armida Mucci, Paola Rucci, Francesco Brando, Edoardo Caporusso, Luigi Giuliani, Antonio Melillo, Pasquale Pezzella, Paola Bucci, Paola Rocca, Alessandro Rossi, Alessandro Bertolino, Rodolfo Rossi, Giulio Pergola, Silvana Galderisi, Mario Maj, Italian Network for Research on Psychoses

**Affiliations:** 1 Department of Psychiatry, University of Campania “Luigi Vanvitelli”, Naples, Italy; 2 Department of Biomedical and Neuromotor Sciences, University of Bologna, Bologna, Italy; 3 Department of Neuroscience, Section of Psychiatry, University of Turin, Turin, Italy; 4 Section of Psychiatry, Department of Biotechnological and Applied Clinical Sciences, University of L’Aquila, L’Aquila, Italy; 5 Department of Basic Medical Science, Neuroscience and Sense Organs, University of Bari “Aldo Moro”, Bari, Italy; 6 Department of Systems Medicine, University of Rome Tor Vergata, Rome, Italy

**Keywords:** BNSS, cognition, five-factor model, functional capacity, hierarchical model, negative symptoms

## Abstract

**Background:**

The conceptualization of negative symptoms (NS) in schizophrenia is still controversial. Recent confirmatory factor-analytic studies suggested that the bi-dimensional model (motivational deficit [MAP] and expressive deficit [EXP]) may not capture the complexity of NS structure, which could be better defined by a five-factor (five NS domains) or a hierarchical model (five NS domains as first-order factors, and MAP and EXP, as second-order factors). A validation of these models is needed to define the structure of NS. To evaluate the validity and temporal stability of the five-factor or the hierarchical structure of the brief negative symptom scale (BNSS) in individuals with schizophrenia (SCZ), exploring associations between these models with cognition, social cognition, functional capacity, and functioning at baseline and at 4 years follow-up.

**Methods:**

Clinical variables were assessed using state-of-the-art tools in 612 SCZ at two-time points. The validity of the five-factor and the hierarchical models was analyzed through structural equation models.

**Results:**

The two models had both a good fit and showed a similar pattern of associations with external validators at the two-time points, with minor variations. The five-factor solution had a slightly better fit. The associations with external validators favored the five-factor structure.

**Conclusions:**

Our findings suggest that both five-factor and hierarchical models provide a valid conceptualization of NS in relation to external variables and that five-factor solution provides the best balance between parsimony and granularity to summarize the BNSS structure. This finding has important implications for the study of pathophysiological mechanisms and the development of new treatments.

## Introduction

Negative symptoms (NSs) are a core component of schizophrenia psychopathology, contributing significantly to low remission rates, poor response to pharmacological and psychosocial interventions, diminished quality of life, and impaired functional outcomes [1–9]. For these reasons, these symptoms continue to represent a formidable challenge in the clinical management of individuals suffering from schizophrenia [10–17].

In particular, negative symptoms have been shown to have a direct effect on functioning, especially in the “interpersonal relationships” domain, independent of other predictors such as neurocognition and functional capacity. Furthermore, these symptoms have also an indirect effect, through social competence, on various domains of functioning, including “interpersonal relationships,” “work skills,” and “everyday life skills” [18–20]. A meta-analysis found that negative symptoms mediate the relationship between neurocognition and functional outcomes [21]. However, a notable limitation in much of this research is the assessment of negative symptoms as a singular, unitary dimension. Moreover, many studies investigating the associations with functioning and neurocognition used rating scales, such as the scale for the assessment of negative symptoms (SANS) [22] or the positive and negative syndrome scale (PANSS) [23], which are misaligned with the current conceptualization of negative symptoms and include items assessing neurocognition or disorganization [1].

Negative symptoms represent a complex and heterogeneous psychopathological dimension, including different constructs. Specifically, according to the consensus statement on negative symptoms, provided within the NIMH-Measurement and Treatment Research to Improve Cognition in Schizophrenia (MATRICS) initiative [24], these symptoms are categorized as follows: (a) avolition; (b) asociality; (c) anhedonia; (d) blunted affect, and (e) alogia.

Two second-generation clinician-rated scales were developed after the MATRICS consensus statement and are now regarded as the gold standard instruments for evaluating negative symptoms in individuals with schizophrenia: the brief negative symptom scale (BNSS) [25] and the clinical assessment interview for negative symptoms (CAINS) [26]. These scales address limitations inherent in first-generation rating scales, such as the SANS or the PANSS.

Different exploratory factor analytic studies, conducted with first-generation (PANSS, SANS) or second-generation rating scales (BNSS, CAINS), have consistently substantiated the multidimensional nature of negative symptoms [1, 10]. Historically, the prevailing structural model has been a two-factor construct, which categorizes negative symptoms into two domains: the motivational deficit domain (MAP), consisting of avolition, anhedonia, and asociality, and the expressive deficit domain (EXP), consisting of blunted affect and alogia [1, 10, 27]. This model is consistent with the observation that the two negative symptom domains are associated with different behavioral and neurobiological correlates, as well as with different clinical and social outcomes [1–4, 6, 10, 28–39]. In particular, the MAP domain is associated with deficits in different aspects of motivation [1–4, 6, 10, 33–35, 40–42], or general impairment in decision making and executive control of behavior, although this latter hypothesis needs further investigations [28]. The EXP domain, on the other hand, is associated with impairments in cognition and social cognition and might be subtended by a diffuse neurodevelopmental alteration in brain connectivity leading to deficits in overall cognition [1–4, 6, 10, 30, 43, 44].

In terms of functional outcomes, research suggests that the MAP domain is associated with more pronounced impairment in functioning than the EXP domain [2, 3, 6]. MAP appeared to have both direct and indirect effects on functional outcome, particularly in the area of “interpersonal relationships”, whereas EXP was only indirectly and weakly related to “everyday life skills” [2, 6]. Within a network model, MAP showed strong associations with “interpersonal relationships” and “work skills,” whereas EXP was associated with “everyday life skills” [3, 4], which in turn was associated with functional capacity.

The two-factor solution of negative symptoms has been very influential over time, guiding the way research studies have been conducted to investigate the pathophysiological mechanisms of negative symptoms [30], the way rating scales are scored in statistical analyses, and the way clinical trials have been designed to develop treatments for these symptoms.

However, more recently, multicenter studies [45–50], using confirmatory factor analysis (CFA) [45, 47–50] or network analysis [3, 46, 51], have questioned the validity of the two-factor model and reported a more complex structure of negative symptoms. Cumulatively, their results indicate that a five-factor model, aligning with the five individual negative symptoms delineated by the NIMH-MATRICS Consensus statement, and a hierarchical model (with five individual negative symptoms as first-order factors, and the MAP and EXP domains as second-order factors) yielded a superior fit compared to the traditional two-factor solution. A more faithful representation may be achieved through the delineation of the five NIMH consensus individual negative symptoms. This re-conceptualization could have significant implications for the identification of neurobiological underpinnings and the development of novel treatment strategies for negative symptoms. Nevertheless, current evidence supporting distinct neurobiological correlates for each of the five individual negative symptoms remains preliminary, underscoring the need for further targeted investigation [52, 53].

Therefore, in light of the above observations, it would be of great interest to examine the comparative fit of the most recently endorsed negative symptom models (five-factor and hierarchical models) by testing their comparative association with independent measures such as cognitive impairment, functional capacity, and various aspects of real-life functioning. Previous investigations have been carried out on this topic. In particular, Ang et al. [50] found that MAP and two of its component symptoms (avolition and asociality), assessed with the BNSS, showed a significant negative correlation with the global assessment of functioning scores, while the EXP and its component symptoms were unrelated with functioning measures. Moreover, Ahmed et al. [54] used a structural equation model (SEM) analysis to examine the comparative external validity of four negative symptom models with cognition, functioning, and psychopathology. They found that the five-factor and the hierarchical factor model provided the best fits to the data.

However, the two above-mentioned studies did not examine the associations of individual negative symptoms with social cognition and functional capacity, which, as mentioned above, have been shown to have a high correlation with negative symptoms [2–4, 6] in pathways to functional outcome, nor did they examine the associations with external validators over time, looking at the potential stability of the same associations.

Therefore, the present study aims to fill this gap by examining the comparative external validity of the five-factor model and the hierarchical model with respect to neurocognition, social cognition, functioning, and functional capacity at baseline and at 4 years of follow-up. We did not include as validators depression or other psychopathological dimensions, or extrapyramidal side effects, which may be confounding factors causing secondary negative symptoms [1], or have an independent impact on functional outcome, because we were interested in investigating the comparative validity of the two-factor model and hierarchical model of both primary and secondary negative symptoms, in relation to outcome measures. Other publications [4, 6] have reported on all determinants of functioning in the cohort of subjects included in the present analysis, which does not involve any of the previously published data or analyses.

## Methods

### Participants

The present study has been conducted in the framework of the Italian Network for Research on Psychoses [2–4, 6] project.

Study participants consisted of community-dwelling patients with schizophrenia (SCZ) who had been stabilized with antipsychotic treatment. Participants were consecutively recruited at the outpatient units of 26 Italian university psychiatric clinics and/or mental health departments between March 1, 2012, and September 30, 2013. Inclusion criteria were a diagnosis of schizophrenia according to DSM-IV, confirmed with the Structured Clinical Interview for DSM IV — Patient version (SCID-I-P), and an age between 18 and 65 years. Exclusion criteria were (a) history of head trauma with loss of consciousness; (b) history of moderate to severe mental retardation or neurological diseases; (c) history of alcohol and/or substance abuse in the last 6 months; (d) current pregnancy or lactation; (e) inability to provide informed consent; and (f) treatment modifications and/or hospitalization due to symptom exacerbation in the last 3 months.

All patients recruited by those participating centers at baseline were invited to participate in the follow-up study 4 years later. Of the 26 Italian university psychiatric clinics or mental health departments involved in the cross-sectional investigation [2, 3], 24 joined the follow-up study [4, 6]. Exclusion criteria for the follow-up study were (a) history of head trauma with loss of consciousness in the 4-years interval between baseline and follow-up; (b) progressive cognitive deterioration possibly due to dementia or other neurological illness diagnosed in the last 4 years; (c) history of alcohol and/or substance abuse in the last 6 months; (d) current pregnancy or lactation; (e) inability to provide informed consent; and (f) treatment modifications and/or hospitalization due to symptom exacerbation in the last 3 months.

The baseline and follow-up studies were performed in accordance with the ethical standards laid down in the 1964 Declaration of Helsinki and were approved by the Ethics Committee of the involved institutions. All participants signed a written informed consent to participate after receiving a detailed explanation of the study’s procedures and goals.

### Assessment instruments

At baseline, socio-demographic variables such as age, education, and gender were collected.

Negative symptoms were assessed with the Brief Negative Symptom Scale, validated in Italian by Mucci and colleagues [25, 55]. The scale comprises 13 items, organized into six subscales (five negative symptom subscales: anhedonia, asociality, avolition, blunted affect, and alogia, and a control subscale: lack of distress). All the items are rated on a 7-point (0–6) scale, thus ranging from absent (0) to moderate (3) to extremely severe (6).

The measurement and treatment research to improve cognition in schizophrenia (MATRICS) consensus cognitive battery (MCCB) [56, 57] was used for the assessment of the following neurocognitive domains: speed of processing, verbal memory and learning, visual memory and learning, reasoning and problem solving, attention and vigilance, and working memory. Higher scores on all domains reflect better neurocognitive function in the corresponding domains.

Social cognition was assessed through the facial emotion identification task (FEIT) [58] and the awareness of social inference test (TASIT) [59]. FEIT is a facial emotion recognition test that consists of identifying the correct emotion (joy, anger, fear, disgust, surprise, sadness, or neutral) represented in a specific photo. A total of 55 photos are presented randomly [58]. The total test score was computed as the number of correct answers. TASIT [59] consists of seven scales (positive emotions, negative emotions, sincere, simple sarcasm, paradoxical sarcasm, sarcasm enriched, and lie), organized into 59 videos divided into three sections (TASIT 1–3): TASIT 1 “The Emotion Evaluation Test,” which explores emotional processing; TASIT 2 “Social Inference-Minimal,” and TASIT 3 “Social Inference-Enriched,” which explore theory of mind. Higher scores on TASIT reflect better social cognition skills.

Real-life functioning was evaluated using the specific level of functioning (SLOF) scale, a hybrid instrument that evaluates many aspects of functioning and is based on the key caregiver’s judgment on the behavior and functioning of the patient [60]. It consists of 43 items arranged into the following domains: physical efficiency, skills in self-care, interpersonal relationships, social acceptability, everyday life skills, and work skills. In our study, the scale was administered by a trained researcher to a key relative of each patient. Only the domains interpersonal relationships, work skills, and everyday life skills were used, as the other subscales showed ceiling effects. Each of the items is rated on a 5-point Likert scale (1 = poorest functioning, 5 = best functioning).

Functional capacity was evaluated using the short version of the University of California San Diego (UCSD) performance-based skills assessment brief (UPSA-B) [61], a performance-based instrument that assesses “financial skills” (e.g., counting money and paying bills) and “communication skills” (e.g., to dial a telephone number for emergency or reschedule an appointment by telephone). The total score, ranges from 0 to 100, with higher score reflecting higher functional capacity.

At follow-up, a clinical form was filled with data about the course of the disease and treatment information during the previous 4 years, using every available source of information (patients, relatives, medical records, and mental health workers). All baseline assessments were also conducted at follow-up, using the same assessment tools.

### Statistical analysis

We estimated and compared structural equation models (SEM) that included the negative symptom domains and the external variables neurocognition, social cognition, functioning, and functional capacity. Variables included as external validators are shown in Table 1. Results were expressed as standardized regression coefficients. Coefficients between 0.10 and 0.29 in absolute value were interpreted as weak linear associations, from 0.30 to 0.49 as moderate associations, and from 0.50 to 1 as strong associations, using Cohen’s criteria to indicate small, medium, and large effects [62, 63].

**Table 1.** External validation variables

Variable	Assessment scale, subtest/domain
Neurocognition	MCCB NAB – Reasoning and problem solving
	MCCB CPT_IP – Attention and vigilance
	MCCB BVMT_R – Visual memory and learning
	MCCB HVLT_R – Verbal memory and learning
	MCCB TMT A-BACS SC – Category fluency and Processing speed
	MCCB WMS-III SS-LNS – Working memory
Social cognition	TASIT_Sect1
	TASIT_Sect2
	TASIT_Sect3
	FEIT
Functioning	SLOF – Everyday life skills
	SLOF – Interpersonal Relationships
	SLOF – Work Skills
Functional capacity	UPSA_B

Abbreviations: BACS SC, brief assessment of cognition in schizophrenia symbol coding; BVMT-R, brief visuospatial memory test-revised; CPT-IP, continuous performance test, identical pairs; FEIT, facial emotion identification test; HVLT-R, Hopkins verbal learning test-revised; LNS, letter-number span; NAB, neuropsychological assessment battery; SLOF, specific levels of functioning; TASIT, the awareness of social inference test; TMT, trail making test-part A; UPSA-B, UCSD performance-based skills assessment; WMS-III SS, Wechsler memory scale spatial span.

SEM combines factor analytic models and structural regression paths that depict association among latent and observed variables. For each external variable, we estimated two structural models of negative symptoms, the five-factor model that considers the five individual negative symptoms as separate domains and the hierarchical model that includes the five individual negative symptom domains as first-order factors, and MAP and EXP domains as second-order factors. We focused on these two models because they proved to be the best factor solutions identified by Ahmed et al. [54] across 5 studies in terms of goodness of fit and external validity. The five-factor and the hierarchical models were estimated separately on baseline and follow-up data. We designated each external variable as a latent variable, except for the functional capacity that is measured by a single variable.

Model fit was evaluated using indices of absolute fit, including the comparative fit index (CFI), the Tucker–Lewis index (TLI), the root mean square error of approximation (RMSEA), and the standardized root mean square residual (SRMR). The CFI and TLI are incremental fit indices that compare the independence model with the hypothesized model [64]. The SRMR is a residual-based index of the difference between sample and hypothesized variance–covariance matrices. The RMSEA is a parsimony index that evaluates the fit between the hypothesized model and the population covariance matrix [65]. Evidence of model fit was determined according to standard interpretations of the fit indices, including CFI and TLI values of at least 0.950, and an RMSEA no greater than 0.080 [66]. The SRMR values range from 0 to 1, with values of 0.080 or lower indicative of good-fitting models.

Information criteria including the Akaike information criterion (AIC), Bayesian information criteria (BIC), and the sample size–adjusted Bayesian information criteria were used to evaluate the relative fit of nested models [67]. These information criteria can only be interpreted in a comparison between models, with lower values indicating better model fit [67]. Mplus software (version 7.3; Muthén and Muthén) was used to conduct these analyses.

## Results

### Sample characteristics

Of the 921 patients who participated in the study at baseline, 618 patients provided follow-up data, and 612 with complete baseline and follow-up BNSS data were included in the present study. Patients were predominantly male, *N* = 422 (69%) versus *N* = 190 women [31%] and had a mean age of 45 years (SD = 10.5) at follow-up. Supplementary Tables S1 and S2 provide the demographic and clinical characteristics of the study sample, as well as the descriptive statistics of the external variables. Table 2 lists the BNSS items used in the study and their mean and standard deviation at baseline and follow-up. All item scores decreased significantly from baseline to follow-up.

**Table 2.** Mean and standard deviation of BNSS items at baseline and follow-up

		Baseline		Follow-up	
BNSS domains and items	*N*	Mean	SD	Mean	SD
Anhedonia					
BNSS1	612	2.86	1.54	2.54	1.57
BNSS2	612	2.96	1.57	2.55	1.56
BNSS3	612	2.86	1.58	2.61	1.62
Asociality					
BNSS5	612	3.28	1.57	2.88	1.60
BNSS6	612	3.01	1.59	2.76	1.58
Avolition					
BNSS7	612	2.87	1.61	2.59	1.61
BNSS8	612	2.82	1.61	2.59	1.57
Blunted affect					
BNSS9	612	2.71	1.67	2.61	1.59
BNSS10	612	2.64	1.77	2.53	1.66
BNSS11	612	2.69	1.79	2.54	1.65
Alogia					
BNSS12	612	2.21	1.72	2.06	1.69
BNSS13	612	2.42	1.79	2.27	1.73

Abbreviations: BNSS, brief negative symptom scale; BNSS items: 1 = intensity of pleasure during activities; 2 = frequency of pleasurable activities; 3 = intensity of expected pleasure from future activities; 5 = asociality behavior; 6 = asociality internal experience; 7 = avolition behavior; 8 = avolition internal experience; 9 = facial expression; 10 = vocal expression; 11 = expressive gestures; 12 = quantity of speech; 13 = spontaneous elaboration.

*Note:* All item scores decreased significantly (Wilcoxon’s test, *p* < 0.001) from baseline.

### Model fit

The external validation of the BNSS five-factor and hierarchical models indicated that the factor solutions had an excellent fit in the confirmatory SEM models (Table 3). Specifically, all absolute fit indices CFI and TLI were >0.95, and RMSEA and SRMR were <0.08. Therefore, we selected the model based on lower values of the comparative fit index AIC.

**Table 3.** Goodness of fit indices of SEM models at baseline (A) and follow-up (B)

Domain	Model	CFI	TLI	RMSEA	SRMR	AIC	BIC	aBIC
(A)								
Neurocognition								
	**Five-factor**	**0.980**	**0.975**	**0.052**	**0.024**	**35946.693**	**36252.121**	**36033.058**
	Hierarchical (second-order five-factor)	0.976	0.971	0.056	0.035	35984.238	36258.680	36061.841
Social cognition								
	**Five-factor**	**0.975**	**0.965**	**0.070**	**0.043**	**31668.861**	**31934.450**	**31743.961**
	Hierarchical (second-order five-factor)	0.974	0.966	0.069	0.045	31675.887	31910.491	31742.225
Functioning								
	**Five-factor**	**0.976**	**0.967**	**0.066**	**0.029**	**36329.869**	**36608.738**	**36408.724**
	Hierarchical (second-order five-factor)	0.972	0.965	0.067	0.039	36353.979	36601.863	36424.073
Functional capacity								
	**Five-factor**	**0.982**	**0.973**	**0.070**	**0.017**	**25355.430**	**25590.034**	**25421.769**
	Hierarchical (second-order five-factor)	0.977	0.969	0.074	0.027	25390.930	25594.548	25448.506
(B)								
Neurocognition								
	**Five-factor**	**0.988**	**0.985**	**0.042**	**0.020**	**34660.310**	**34965.064**	**34746.004**
	Hierarchical (second-order five-factor)	0.987	0.984	0.043	0.026	34670.453	34944.290	34747.453
Social cognition								
	**Five-factor**	0.980	0.972	0.067	0.037	30256.328	30521.332	30330.845
	Hierarchical (second-order five-factor)	**0.980**	**0.975**	**0.064**	**0.039**	**30248.680**	**30482.767**	**30314.503**
Functioning								
	**Five-factor**	**0.975**	**0.967**	**0.069**	**0.040**	**34715.893**	**34994.147**	**34794.136**
	Hierarchical (second-order five-factor)	0.972	0.966	0.071	0.046	34737.941	34985.278	34807.489
Functional capacity								
	**Five-factor**	0.988	0.981	0.061	0.015	24269.907	24503.994	24335.730
	Hierarchical (second-order five-factor)	**0.987**	**0.983**	**0.059**	**0.018**	**24269.433**	**24472.602**	**24326.562**

Abbreviations: aBIC, sample size adjusted BIC; AIC, Akaike information criterion; BIC, Bayesian information criterion; CFI, comparative fit index; RMSEA, root mean square error of approximation; SRMR, standardized root mean squared residual; TLI, Tucker–Lewis index.

*Note:* The preferred model for each clinical domain is in boldface.

At baseline, the five-factor solution proved to be better, as compared to the hierarchical model, for all external variables (neurocognition, social cognition, functional capacity, and functioning).

At follow-up, the five-factor solution proved to be better, as compared to the hierarchical model, for neurocognition and functioning, while the hierarchical solution was better for social cognition and functional capacity.

### Association of negative symptom domains with clinical variables in the SEM models

#### Baseline

As shown in Table 4, at baseline MAP showed a significant negative moderate association with functioning (β = −0.303, *p* = 0.003), while EXP had a significant moderate negative association with functional capacity (β = −0.404, *p* < 0.001). Regarding individual negative symptoms, alogia showed moderate negative associations with neurocognition (β = −0.444, *p* < 0.001), social cognition (β = −0.336, *p* < 0.001), and functional capacity (β = −0.398, *p* < 0.001).

**Table 4.** Path coefficients of structural models depicting associations with clinical external variables at baseline (A) and follow-up (B)

BNSS factors	Neurocognition		Social cognition		Functioning		Functional capacity	
	Estimate^a^	*p*	Estimate^a^	*p*	Estimate^a^	*p*	Estimate^a^	*p*
(A)								
MAP	−0.010	0.925	0.084	0.415	**−0.303**	**0.003**	0.128	0.201
EXP	*−0.251*	*0.019*	*−0.269*	*0.009*	−0.192	0.057	**−0.404**	**<0.001**
Anhedonia	0.128	0.089	−0.036	0.635	−0.041	0.602	*0.215*	*0.002*
Asociality	0.038	0.737	0.179	0.12	−0.062	0.608	−0.083	0.433
Avolition	*−0.239*	*0.054*	−0.146	0.242	−0.205	0.112	−0.089	0.437
Blunted affect	*0.205*	*0.026*	0.112	0.226	−0.060	0.529	0.039	0.647
Alogia	**−0.444**	**<0.001**	**−0.336**	**<0.001**	−0.164	0.033	**−0.398**	**<0.001**
(B)								
MAP	0.053	0.602	0.013	0.902	**−0.331**	**0.001**	0.104	0.277
EXP	**−0.516**	**<0.001**	**−0.384**	**<0.001**	−0.242	0.011	**−0.542**	**<0.001**
Anhedonia	−0.080	0.249	−0.096	0.172	−0.139	0.045	−0.069	0.288
Asociality	**0.386**	**<0.001**	0.203	0.043	−0.073	0.466	0.134	0.153
Avolition	**−0.350**	**0.002**	−0.204	0.078	−0.133	0.245	−0.074	0.491
Blunted affect	*−0.186*	*0.047*	0.146	0.125	−0.106	0.256	−0.168	0.056
Alogia	*−0.248*	*0.001*	**−0.471**	**<0.001**	−0.168	0.029	**−0.3**	**<0.001**

Abbreviations: EXP, expressive deficit domain; MAP, motivational deficit domain.

*Note*: Moderate to strong associations (≥0.30) are shown in boldface.

a Standardized coefficient.

Other weak associations emerged: EXP with neurocognition and social cognition, blunted affect with neurocognition, alogia with functioning, and anhedonia with functional capacity.

#### Follow-up

As shown in Table 4, at follow-up MAP showed a moderate negative association with functioning (β = −0.331, *p* = 0.001), while EXP had strong negative associations with neurocognition (β = −0.516, *p* < 0.001) and functional capacity (β = −0.542, *p* < 0.001) and moderate association with social cognition (β = −0.384, *p* < 0.001). Regarding individual negative symptoms, asociality (β = 0.386, *p* < 0.001) and avolition (β = − 0.350, *p* = 0.002) showed moderate associations with neurocognition. Furthermore, alogia showed moderate associations with social cognition (β = −0.471, *p* < 0.001) and functional capacity (β = −0.3, *p* < 0.001) and a weak association with neurocognition (β = −0.248, *p* = 0.001).

Other weak associations were found of EXP, anhedonia, and alogia with functioning, asociality with social cognition, and blunted affect with neurocognition.

## Discussion

In this paper, we utilized SEM to investigate the external validity of both the five-factor model and the hierarchical model of the BNSS, in relation to cognition, social cognition, functioning, and functional capacity at baseline and at a 4-year follow-up.

Consistent with recent multicenter studies, our results confirmed the validity of the five-factor (anhedonia, avolition, asociality, blunted affect, and alogia) and the hierarchical model (five individual negative symptoms as first-order factors, and the two domains, MAP and EXP, as second-order factors) of negative symptoms [45–47, 49, 50, 54].

Based on the included external validators, these models proved to be equivalent in terms of fit to the data both at baseline and follow-up. Both models demonstrated a commendable fit at both baseline and follow-up. At baseline, the five-factor model exhibited a slight advantage over the hierarchical model across all evaluated external validators. In contrast, at follow-up, the hierarchical model was modestly better than the five-factor structure, particularly concerning social cognition and functional capacity. Nonetheless, the differences in the CFI, TLI, and AIC values between the two models were minimal. As such, these slight variations in the negative symptoms structure are negligible in terms of potential clinical implications.

Regarding the relationship of the two BNSS models with external variables, we found similar patterns of associations at the two time points despite minor variations, including the stronger association, at both time points, of the EXP with neurocognition and functional capacity and of MAP with real-life functioning. However, within the wider dimensions of EXP and MAP, only some items follow the same pattern of associations, suggesting that the five-factor solution provides the best balance between parsimony and granularity to summarize BNSS structure.

Indeed, within MAP, the component domains (anhedonia, asociality, and avolition) did not show an association with functioning at baseline, and at follow-up, asociality and avolition were associated with neurocognition, although in different directions (which may explain why the association is not found for the MAP factor). Furthermore, within the EXP, alogia, but not blunted effect, was associated with neurocognition and social cognition.

It is particularly compelling to note the shift in associations from baseline to follow-up, as delineated in Table 4. For instance, EXP’s associations became stronger, most notably with neurocognition and functional capacity, progressing from moderate to strong negative associations. This suggests that, over time, the effect of EXP on neurocognitive deficits and functional capacity might be more pronounced than initially observed. Equally intriguing is the emergence of associations of individual negative symptoms, such as asociality and avolition, with neurocognition. Meanwhile, alogia maintained its significant associations with social cognition and functional capacity but exhibited a weaker relationship with neurocognition.

These findings suggest that the use of the BNSS two-factor model may lead to a loss of information or mask associations of the five NIMH consensus individual negative symptoms (anhedonia, asociality, avolition, blunted affect, and alogia), which may represent distinct constructs underlying different behavioral and pathophysiological processes. However, the broader factors of MAP and EXP may have a more stable pattern of associations, albeit at the expense of information, as they include multiple items, whereas the individual domains have a suboptimal number of items.

Our results concerning the relationships of negative symptoms with neurocognition are only partially consistent with those of Ahmed et al. [54]. In particular, while Ahmed et al. found a negative relationship of MAP, avolition, and blunted affect with neurocognition, our findings indicated a negative association between EXP and alogia with neurocognition at both time points. This stands in contrast with Ahmed’s results. Notably, in our study, the negative association between avolition and neurocognition emerged only at the follow-up assessment. A potential explanation for these discrepancies could be the small sample size in Ahmed’s study (*N* = 146), which was recruited from two countries and may have led to unstable estimates.

As to functioning, we found that patients exhibiting higher levels of MAP consistently demonstrated poorer functioning at both time points. We observed weak relationships between functioning, EXP, alogia, and anhedonia. This result is consistent with the evidence suggesting that MAP is associated with a greater impairment in functioning, as compared to the EXP domain, in particular in the area of “interpersonal relationships” and “work skills” [2–4, 6]. Nevertheless, when comparing our results with those from Ahmed et al. [54], discrepancies emerge, as they reported a negative relationship between functioning not only with MAP, but also with anhedonia, avolition, and blunted affect. These differences may be attributed to varying sample sizes between studies and the use of different tools to assess functioning.

Our results concerning the negative association of EXP and alogia with social cognition and functional capacity at both time points are original and cannot be compared with other findings from the literature with comparable methodology and tools. However, regarding the relationship between social cognition and EXP, this result can be interpreted in the light of one of the main theories of causation of EXP and its component symptoms, which poses at the basis of this domain deficits in emotion identification and discrimination and, more in general, abnormalities in perception of nonverbal social cues [28], with a consequent inability to infer meaning from social situations and behaviors and to respond appropriately. In addition, the result of the association between functional capacity and EXP/alogia can be interpreted in the light of previous findings of a direct [68] or indirect (mediated by everyday life skills) relationship [3, 4] between functional capacity and EXP. Finally, it is also important to note that the functional capacity serves as a linking mechanism between neurocognition/social cognition and the “everyday life skills” domain of functioning, which, in turn, is related to EXP [3, 4]. Therefore, overall, the associations between EXP and alogia with cognition (neurocognition and social cognition) and functional capacity might be interpreted in the light of very complex interconnections between these factors.

While our study provides valuable insights into the negative symptom structure and its association with cognition and functional outcomes, some limitations should be acknowledged: (i) our sample is certainly representative of Italian community-dwelling patients with stable and chronic schizophrenia but cannot provide information on what happens in the early stages of the disease. Therefore, further studies including patients at the onset of psychoses are encouraged; (ii) while we can identify associations between negative symptoms and external validators, we cannot infer causality. Further studies would be required to understand the temporal evolution and causal relationships between negative symptoms and functional outcomes.

In conclusion, the five-factor and the hierarchical models provide an optimal conceptualization of negative symptoms in relation to external variables. The pattern of associations with external variables of the two models at the two-time points, despite minor variations, suggests that the five-factor solution provides the best balance between parsimony and granularity to summarize BNSS structure. In fact, when we looked at associations of broader BNSS MAP/EXP domains and of individual negative symptoms with external variables, we found a different pattern of associations with the possibility that broader MAP/EXP domains might mask significant associations with one or more of the narrower symptoms.

These results have important implications for research and clinical practice.

In fact, given the validity of the two models and the presence of domain-specific associations between first-order dimensions and external variables, we can conclude that the five domains of negative symptoms (5 individual negative symptoms) are distinct. In this direction, the current DSM-5 description of negative symptoms should move from the broad MAP/EXP domains to the five negative symptom domains. Clinicians should avoid the use of first-generation rating scales such as PANSS and SANS as they do not adequately capture the five negative symptom domains or the MAP/EXP domains; the use of second-generation rating scales such as BNSS and CAINS should be encouraged as these scales are able to capture the complexity of negative symptoms (both the five-factor and the hierarchical structure) [1, 10]. In addition, clinical trials and research studies on negative symptoms, investigating their response to treatment or their pathophysiological bases, should have as a primary outcome the two negative symptom domains (EXP/MAP) and then, they should move on the level of the five negative symptom domains [54].

Finally, the correct conceptualization of negative symptoms, if implemented in research and clinical practice, could allow the identification of pathophysiological mechanisms or new treatment strategies specific to one or more negative symptoms, which would be precluded or delayed by the adoption of previous negative symptom factor models.

## Supporting information

Giordano et al. supplementary material Giordano et al. supplementary material

## Data Availability

The original contributions presented in the study are included in the article, further inquiries can be directed to the corresponding author/s.

## Supplementary material

The supplementary material for this article can be found at https://doi.org/10.1192/j.eurpsy.2023.2478.
