# Supplementary material for: External validation of the five domains of negative symptoms: Focus on cognition, functional capacity, and real-world functioning
Source: Eur Psychiatry. 2023 Dec 15;67(1):e3. doi: 10.1192/j.eurpsy.2023.2478 (PMC10964270; doi:10.1192/j.eurpsy.2023.2478)
Supplement: Giordano et al. supplementary material [file S0924933823024781sup001.docx]

**The Brief Negative Symptom Scale: external validation of symptom domains with cognitive and functioning-related variables in subjects with schizophrenia**

**Index**

**Supplementary Table 1: Characteristics of study participants at follow-up.**

**Supplementary Table 2: Descriptive statistics of external validators**

**Supplementary Table 1: Characteristics of study participants at follow-up (N=612).**

| **Variables** | **Percentage frequency or mean±SD** |
| --- | --- |
| **Gender (% males)** | 69.0 |
| **Age (years, mean±SD)** | 45.1±10.5 |
| **Married (%)** | 7.5 |
| **Working (%)** | 34.4 |
| **Education (years, mean±SD)** | 11.7±3.4 |
| **Stable affective relationships (%)** | 18.9 |
| **Legal problems (%)** | 1.3 |
|  |  |
| **Current drug treatment (%)** | 99.2 |
| *antipsychotics (%)* | 98.8 |
| *first generation* | 13.1 |
| *second generation* | 73.6 |
| *first and second generation* | 13.3 |
| *antidepressants (%)* | 17.6 |
| *mood stabilizers (%)* | 26.0 |
| *anxiolytics (%)* | 32.8 |
| *anticholinergics (%)* | 9.5 |
|  |  |
| **Polypharmacy (%)** | 53.8 |
|  |  |
| **Any psychosocial interventions (%)** | 34.3 |
|  |  |
| **Psychotherapy (%)** | 14.9 |
|  |  |
| **Substance abuse (%)** | 4.9 |
| **Alcohol abuse (%)** | 4.6 |
| **Smoking (%)** | 42.0 |
| **Suicide attempts (%)** | 3.4 |

*Number of patients with missing data: Working 8; Stable affective relationships 5; Legal problems 3; Current drug treatment 2; Antipsychotics 9; Antidepressants 9; Mood stabilizers 9; Anxiolytics 9; Anticholinergics 9; Polypharmacy 9; Any psychosocial interventions 3; Psychotherapy 6; Substance abuse 1; Alcohol abuse 1; Smoking 9.*

**Supplementary Table 2:** **Descriptive statistics of external validators (N=612).**

| **Characteristic** | **Mean±SD** |
| --- | --- |
| **Baseline** |  |
| **Social cognition** |  |
| **FEIT** | 37.0±8.3 |
| **TASIT_Sect1** | 20.1±4.9 |
| **TASIT_Sect2** | 37.6±10.9 |
| **TASIT_Sect3** | 38.5±11.1 |
| **Functional capacity** |  |
| **UPSA_B** | 67.4±21.6 |
| **Functioning** |  |
| **SLOF interpersonal relationships** | 22.8±5.9 |
| **SLOF everyday life skills** | 46.3±8.3 |
| **SLOF work skills** | 20.4±6.0 |
| **Neurocognition** |  |
| **Reasoning and Problem solving** | 9.8±6.5 |
| **Attention and Vigilance** | 1.7±0.8 |
| **Visual memory and learning** | 16.3±8.7 |
| **Verbal memory and learning** | 19.1±5.4 |
| **Processing Speed** | -1.3±1.1 |
| **Working memory** | -1.1±1.0 |
|  |  |
| **Follow up** |  |
| **Social cognition** |  |
| **FEIT** | 37.3±8.1 |
| **TASIT_Sect1** | 20.4±4.8 |
| **TASIT_Sect2** | 38.6±10.3 |
| **TASIT_Sect3** | 38.8±9.7 |
| **Functional capacity** |  |
| **UPSA_B** | 68.5±23.9 |
| **Functioning** |  |
| **SLOF interpersonal relationships** | 22.2±6.1 |
| **SLOF** **everyday life skills** | 45.2±9.6 |
| **SLOF work skills** | 20.1±6.1 |
| **Neurocognition** |  |
| **Reasoning and Problem solving** | 9.6±6.6 |
| **Attention and Vigilance** | 1.6±0.9 |
| **Visual memory and learning** | 16.1±8.1 |
| **Verbal memory and learning** | 19.6±5.5 |
| **Processing Speed** | -1.3±1.3 |
| **Working memory** | -1.2±1.0 |

*FEIT: Facial Emotion Identification Test; TASIT: The Awareness of Social Inference Test; UPSA-B:UCSD Performance-Based Skills Assessment; SLOF: Specific Levels of Functioning. Number of patients with missing data: SLOF Interpersonal=5; SLOF Everyday life skills=8; SLOF Work skills=5*
